# ﻿Two new species and one new record of the genus *Torodora* Meyrick (Lepidoptera, Lecithoceridae) from China

**DOI:** 10.3897/zookeys.1218.135814

**Published:** 2024-11-22

**Authors:** Shuai Yu, Lin Liu, Xueqing Li, Shuxia Wang

**Affiliations:** 1 College of Agriculture and Biology, Liaocheng University, Liaocheng 252000, China Liaocheng University Liaocheng China; 2 College of Life Sciences, Nankai University, Tianjin 300071, China Nankai University Tianjin China

**Keywords:** Gelechioidea, morphology, taxonomy, Torodorinae

## Abstract

Two new species of the genus *Torodora* Meyrick, 1894 are described from China: *T.lichi***sp. nov.** and *T.mici***sp. nov.** Additionally, *T.silvatica* Park, 2007 is newly recorded for China. Images of the adults and their genitalia are provided.

## ﻿Introduction

*Torodora* Meyrick, 1894 is the largest and type genus of the subfamily Torodorinae (Lecithoceridae). It is distributed in the Oriental, Palearctic, Ethiopian, and Australian region, and comprises more than 230 species (Park et al. 2022). Eighty-eight species of *Torodora* are recorded in China. The genus is characterized by having the following combination of characters: forewing with M_1_ and M_2_ free, M_3_ separated or stalked with R_4+5_, R_4_ and R_5_ stalked; M_1_, M_2_, and M_3_ free; CuA_1_ and CuA_2_ stalked; hindwing with M_2_ present; M_3_ and CuA_1_ stalked or coincident; genitalia morphologically varied and abdominal tergites with zones of spiniform setae. Here, we describe two new species of *Torodora*, as well as the first report of *T.silvatica* Park, 2007 from China.

## ﻿Materials and methods

The specimens were collected at the locations indicated below. Each image was collected using GYZ 450 W high-pressure mercury lamps (Yaming, China). Morphological terminology in the descriptions was in accordance with Gozmány (1978). The wingspan was measured from the tips of the left and right forewings of fully well spread specimens. Slides of genitalia were prepared following Li (2002). Photographs of adults were taken using an M205A stereomicroscope, and photographs of genitalia were taken using a DM750 microscope with Leica Application Suite software version 4.6 (all from Leica, Germany). All photographs were manipulated in Photoshop CC (Adobe, USA).

The examined materials, including the type series of the new species, are deposited at Liaocheng University (**LCU**), Liaocheng, China.

## ﻿Taxonomic accounts

### ﻿ Torodora

Taxon classificationAnimaliaLepidopteraLecithoceridae

Meyrick, 1894

81F4BD02-71D0-5F56-9B82-4EE291109DD3

Torodora Meyrick, 1894: 16. Type species: Torodoracharacteris Meyrick, 1894, by original designation.
Habrogenes Meyrick, 1918: 102. Type species: Habrogeneseupatris Meyrick, 1918, by original designation.
Panplatyceros Diakonoff, 1951: 76. Type species: Panplatycerosserpentina Diakonoff, 1951, by monotypy.
Toxotarca Wu, 1994: 123. Type species: Toxotarcaparotidosa Wu, 1994, by monotypy.

### ﻿ Torodora lichi

Taxon classificationAnimaliaLepidopteraLecithoceridae

Yu & Wang sp. nov.

C6B28598-F396-5BE8-81A9-41B9177708C2

https://zoobank.org/084DF877-08EC-4580-B044-11AFEF6951F6

Figs 1A, B , 2A

#### Type materials.

***Holotype***: China • ♂; Yunnan Prov., Menghai County, Nabanhe; 22.243°N, 100.599°E; 810 m elev.; 2 Aug. 2022; Shuai Yu & Kaijian Teng leg.; slide no. YUS004, in LCU.

#### Diagnosis.

*Torodoralichi* is externally similar to the Thailand’s species, *T.epicharis* Park, 2002. It can be distinguished by the forewing absent of a postmedian line, in the male genitalia by the foot-shaped cucullus, and the juxta with a triangular median process below the posterior margin; *T.epicharis* has a postmedian line in the forewing and has an ovate cucullus and a juxta lacking the median process (Park 2002: 156).

#### Description.

**Figure 1.**
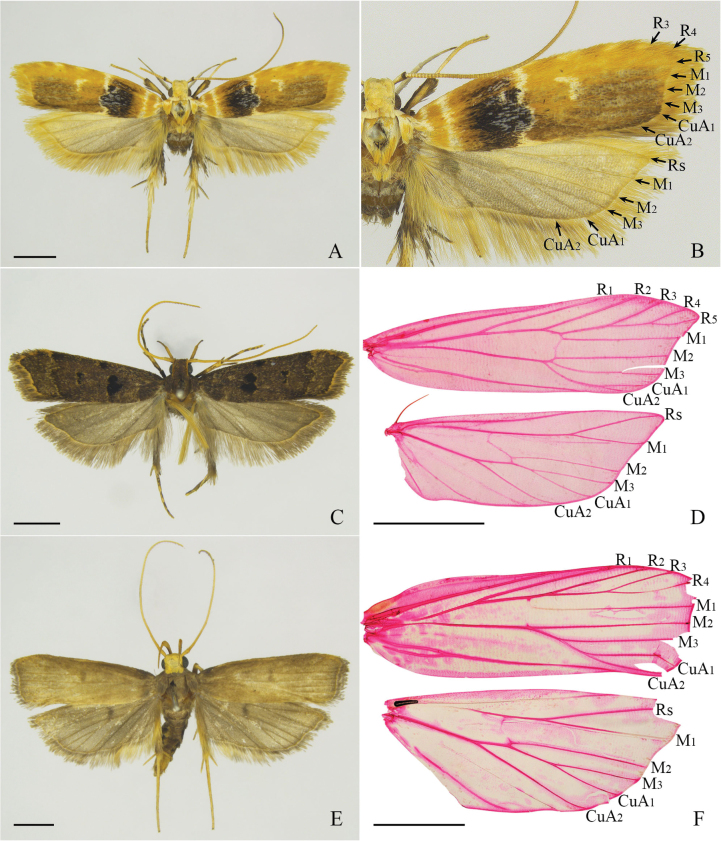
External features and wing venation of *Torodora* spp. **A** external features of *T.lichi* Yu & Wang, sp. nov., holotype, male, slide No. YUS004 **B** right wing venation of *T.lichi* Yu & Wang, sp. nov., holotype, male, slide No. YUS004 **C** external features of *T.mici* Yu & Wang, sp. nov., paratype, male, LCU204 **D** right wing venation of *T.mici* Yu & Wang, sp. nov., paratype, male, LCU033 **E** external features of *T.silvatica* Park, 2007, male, slide No. LCU218 **F** right wing venation of *T.silvatica* Park, 2007, male, slide No. LCU217. Scale bars: 2.0 mm.

Wingspan 15.0 mm (Fig. 1A). Head pale yellow. Antennae pale yellow, scape apically dark brown. Labial palpus with second palpomere white, roughly scaled ventrally; third palpomere white dorsally, dark brown ventrally, as long as second palpomere. Patagium white. Thorax and tegula white, mixed with pale, orange-yellow scales. Forewing with a slightly arched costal margin, apex triangularly produced, termen concave; ground color pale orange-yellow, with three white spots along the distal half of the costal margin; basal 1/4 with two straight, white lines running from the costal margin to the dorsum; a large black patch before the middle, mixed with white scales, anteriorly reaching below the costal margin of the forewing, posteriorly reaching the dorsum; outer margin sinuate; median line white, extending from the costal margin of the forewing along the outer margin of the patch to the dorsum; a crescent pale yellow spot at the distal 2/3 anterior of M_2_; area between median line and termen brown along posterior 2/3; fringe pale orange yellow; venation with R_3_, R_4_, and R_5_ stalked, M_1_, M_2_, and M_3_ free, and CuA_1_ and CuA_2_ stalked. Hindwing and fringe pale, greyish brown; fringe with a pale-yellow basal line; venation with M_3_ and CuA_1_ stalked basally, distant from CuA_2_ at base (Fig. 1B).

**Figure 2.**
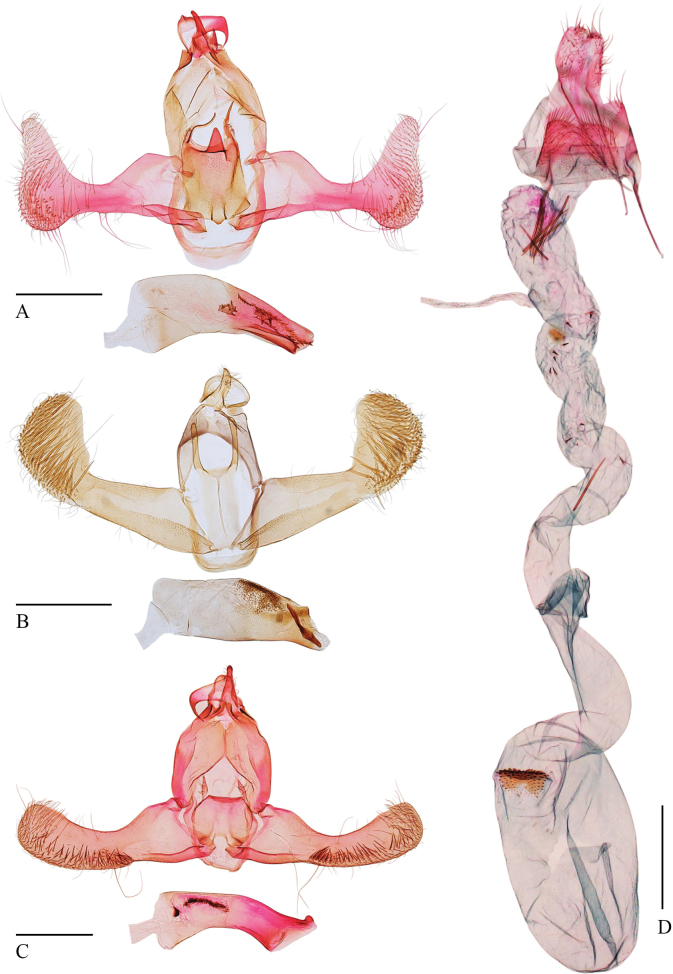
Genitalia of *Torodora* spp. **A** male genitalia of *T.lichi* Yu & Wang, sp. nov., holotype, slide No. YUS004 **B** male genitalia of *T.mici* Yu & Wang, sp. nov., holotype, slide No. LCU034 **C** male genitalia of *T.silvatica* Park, 2007, slide No. LCU217 **D** female genitalia of *T.mici* Yu & Wang, sp. nov., paratype, slide No. LCU219. Scale bars: 0.5 mm.

***Male genitalia*** (Fig. 2A). Uncus elongated and triangular. Gnathos with median process wide at base, slightly narrowed toward the distal 2/5 where it curves and sharply tapers to a pointed apex. Valva wide basally, narrowed medially; cucullus foot-shaped, widened basally, narrowing toward a narrowly rounded apex; outer margin concave, ventral margin round, produced distally; costa deeply concave broadly; sacculus band-shaped. Vinculum U-shaped. Juxta rectangular, longer than wide, with a heavily sclerotized, triangular median process along the posterior margin; posterolateral lobes elongated, horn-shaped; posterior lobes near the posterolateral lobes, weakly sclerotized, digitate, setose; anterior margin with an imbricate process near middle. Phallus wide at the base, gradually narrowing toward a blunt apex, curved; cornuti located distally, consisting of two small spinose plates, with two elongate spiculose bars.

**Female.** Unknown.

#### Distribution.

China (Yunnan Province).

#### Etymology.

The specific epithet is derived from Mandarin *li* (beautiful) and *chi* (wing), referring to the colorful forewing.

### ﻿ Torodora mici

Taxon classificationAnimaliaLepidopteraLecithoceridae

Yu & Wang sp. nov.

50A33528-2F3F-5A61-A7E3-30324F455D59

https://zoobank.org/A7BB5155-311A-4E8E-8106-269201037072

Figs 1C, D , 2B, D

#### Type materials.

***Holotype***: China • ♂; Xizang Autonomous Region [Tibet], Motuo County [Mêdog], Beibengxiang; 29.242°N, 95.171°E; 1239 m elev.; 14 Jun. 2023; Shuai Yu leg.; slide no. LCU034, in LCU. ***Paratypes***: 3 ♂ 1 ♀; same data as holotype; slide nos. LCU033♂, LCU204♂, LCU219♀, in LCU.

#### Diagnosis.

*Torodoramici* is similar to *T.reniformis* Yu & Wang, 2022 in the male genitalia. It can be distinguished by the blackish brown forewing, the juxta reaching near posterior margin of the tegumen, and the cornutus an elongate bar; *Torodorareniformis* has a forewing that is dark brown on the basal 3/4 and orange white on the distal 1/4 (Yu et al. 2022: 16), the juxta reaches far from the posterior margin of the tegumen, and the cornuti consists of needle-like spines (Yu et al. 2022: 24).

#### Description.

Wingspan 13.5–14.0 mm (Fig. 1C). Head dark brown, orange-yellow along lateral surfaces. Antennae yellow. Labial palpus dark brown, distally yellow on second palpomere; third paplomere slender, as long as the second. Thorax and tegula dark brown. Forewing with costal margin nearly straight, slightly curved distally, apex slightly down-curved, termen slightly concave; ground color dark brown, mixed with scattered yellow scales, distal 1/4 of the costal margin yellow; discal stigma rounded, black, outer margin edged with yellow scales; plical stigma nearly rounded, black, anteriorly extending toward discal stigma, outer margin edged with yellow scales; discocellular stigma small, paired, located one above another, with a yellow outer margin; subterminal line yellow, extending from 1/4 of the costal margin sinuated to the distal 1/5 of the dorsum; fringe dark brown, with a yellow basal line; venation with R_1_, R_2_ free, R_3_, R_4_, and R_5_ stalked, R_5_ extending to apex, M_1_, M_2_, M_3_ free, CuA_1_ and CuA_2_ stalked distally. Hindwing greyish brown; fringe greyish brown, with a yellow basal line; venation with M_2_ free, M_3_ and CuA_1_ stalked basally, CuA_2_ distant from M_3_+CuA_1_ at the base (Fig. 1D).

***Male genitalia*** (Fig. 2B). Uncus elongated with widened base. Gnathos with basal plate rounded on posterior margin, median process absent. Valva wide at the base, gradually narrowing to cucullus; cucullus extending obliquely dorsad, apical margin broadly rounded, costal margin nearly straight throughout length, abruptly curved upwards forming inner margin of cucullus; sacculus wide, elongate, densely spiculose. Vinculum U shaped, nearly straight on anterior margin. Juxta rectangular, longer than wide, with a longitudinal median line; posterolateral lobes digitate, reaching near the posterior margin of the tegumen, apex narrowly rounded, setose. Phallus shorter than the valva, straight, uniformly wide basally, narrowing apically; vesica densely granulate; cornutus an elongate bar near apex of vesica.

***Female genitalia*** (Fig. 2D). Eighth abdominal sternite medially concave on posterior margin, forming two lateral parts broadly rounded posteriorly. Apophyses posteriores longer than apophyses anteriores. Antrum cup-shaped and membranous. Ductus bursae nearly wide throughout length, bearing sparse spines; ductus seminalis slender, arising from approximately the posterior 1/4 of ductus bursae, with dense spinules on the inner wall. Corpus bursae elliptical; signum on posterior end, a transverse plate, bearing dense spinules.

#### Distribution.

China (Xizang Autonomous Region [Tibet]).

#### Etymology.

The specific epithet is derived from the Mandarin *mi* (dense) and *ci* (spine), referring to the densely spined sacculus of the male genitalia.

### ﻿ Torodora silvatica

Taxon classificationAnimaliaLepidopteraLecithoceridae

Park, 2007

28ADD6AA-300D-5240-8DA1-4CDF2902EE8B

Figs 1E, F , 2C

Torodora silvatica Park, 2007: 23. Holotype male collected in Thailand, deposited at the University of Osaka Prefecture, Osaka, Japan (OPU).
Thubana silvatica (Park): Park et al. 2013: 312.
Thubana seimaensis Park, 2013: 312. Holotype male collected in Cambodia, deposited at the University of Incheon, Incheon, South Korea (UIK).

#### Specimens examined.

China • 1 ♂; Yunnan Prov., Menghai County, Nabanhe; 22.243°N, 100.599°E; 810 m elev.; 2–3 Aug. 2022; Shuai Yu & Kaijian Teng leg., in LCU • 1 ♂; Yunnan Prov., Jinghong City, Mt. Jinuo; 21.982°N, 100.889°E; 1425 m elev.; 6–7 Aug. 2022; Shuai Yu & Kaijian Teng leg.; slide no. LCU218, in LCU • 1 ♂; Yunnan Prov., Jinghong City, Yexianggu; 22.100°N, 100.520°E; 852 m elev.; 8 Aug. 2022; Shuai Yu & Kaijian Teng leg.; slide no. LCU217, in LCU.

#### Description.

Adult wingspan 16.0–16.5 mm (Fig. 1E).

#### Distribution.

China (Yunnan Province, new record), Cambodia, Thailand.

#### Remarks.

Park (2007) firstly described *T.silvatica* from Thailand, and he later transferred the species to *Thubana* Walker, 1864 (Park et al. 2013). Park et al. (2022) transferred *Th.silvatica* back to *Torodora*. An explanation for this reversal is given: “the venation of forewing differs from that of the type species of *Torodora*, having M_3_ and CuA_1+2_ on a common stalk, as well as that of *Thubana* Walker, but it is no doubt that the forewing color pattern and the male genital characters are closer to the genus *Torodora*” (Park et al. 2022: 368). We follow Park et al.’s treatment and concur with the taxonomic status of *Torodorasilvatica*.

## Supplementary Material

XML Treatment for Torodora

XML Treatment for Torodora lichi

XML Treatment for Torodora mici

XML Treatment for Torodora silvatica

## ﻿Additional information

### Author ORCIDs

Shuai Yu https://orcid.org/0000-0003-3670-2701

Shuxia Wang https://orcid.org/0000-0002-9316-6661

### Data availability

All of the data that support the findings of this study are available in the main text.
